# PulseDB: A large, cleaned dataset based on MIMIC-III and VitalDB for benchmarking cuff-less blood pressure estimation methods

**DOI:** 10.3389/fdgth.2022.1090854

**Published:** 2023-02-08

**Authors:** Weinan Wang, Pedram Mohseni, Kevin L. Kilgore, Laleh Najafizadeh

**Affiliations:** ^1^ Integrated Systems and NeuroImaging Laboratory, Department of Electrical and Computer Engineering, Rutgers University, Piscataway, NJ, United States; ^2^ Department of Electrical, Computer, and Systems Engineering, Case Western Reserve University, Cleveland, OH, United States; ^3^ Department of Physical Medicine & Rehabilitation, Case Western Reserve University and The MetroHealth System, Cleveland, OH, United States

**Keywords:** cuff-less blood pressure estimation, hypertension, photoplethysmography (PPG), electrocardiogram (ECG), arterial blood pressure (ABP), deep learning, dataset, association for the advancement of medical instrumentation (AAMI)

## Abstract

There has been a growing interest in developing cuff-less blood pressure (BP) estimation methods to enable continuous BP monitoring from electrocardiogram (ECG) and/or photoplethysmogram (PPG) signals. The majority of these methods have been evaluated using publicly-available datasets, however, there exist significant discrepancies across studies with respect to the size, the number of subjects, and the applied pre-processing steps for the data that is eventually used for training and testing the models. Such differences make conducting performance comparison across models largely unfair, and mask the generalization capability of various BP estimation methods. To fill this important gap, this paper presents “PulseDB,” the largest cleaned dataset to date, for benchmarking BP estimation models that also fulfills the requirements of standardized testing protocols. PulseDB contains 1) 5,245,454 high-quality 10-s segments of ECG, PPG, and arterial BP (ABP) waveforms from 5,361 subjects retrieved from the MIMIC-III waveform database matched subset and the VitalDB database; 2) subjects’ identification and demographic information, that can be utilized as additional input features to improve the performance of BP estimation models, or to evaluate the generalizability of the models to data from unseen subjects; and 3) positions of the characteristic points of the ECG/PPG signals, making PulseDB directly usable for training deep learning models with minimal data pre-processing. Additionally, using this dataset, we conduct the first study to provide insights about the performance gap between calibration-based and calibration-free testing approaches for evaluating generalizability of the BP estimation models. We expect PulseDB, as a user-friendly, large, comprehensive and multi-functional dataset, to be used as a reliable source for the evaluation of cuff-less BP estimation methods.

## 1. Introduction

Over the past two decades, there has been a growing interest in developing methods to estimate blood pressure (BP) from cardiovascular-related physiological signals, such as the photoplethysmogram (PPG) and/or the electrocardiogram (ECG), that can be continuously acquired using low-cost and disturbance-free sensors. The ultimate goal is that these methods, when implemented on wearable devices, can replace traditional cuff-based approaches (e.g., sphygmomanometer) to enable affordable and continuous BP monitoring.

Recent BP estimation studies have been utilizing data-driven end-to-end deep learning approaches to develop models that learn features from raw physiological signals without relying on prior knowledge (e.g., manually-defined statistic or hemodynamic parameters evaluated from the PPG and ECG signals (1–4)) while offering promising BP estimation accuracy. Examples include combining convolutional neural network (CNN) and recurrent neural network (RNN) (5–7), embedding attention or residual blocks in models (8–10), or incorporating frequency-domain information in addition to the time-domain information (11, 12). Table 1 summarizes 17 most recent deep-learning based BP estimation studies published in recent years.

**Table 1:** Summary of data and BP estimation accuracy information in 17 recent deep learning-based cuff-less blood pressure estimation studies. The error metrics represent the optimal reported testing results.

Reference	Database used	Cleaned dataset information			Reference BP distribution (mmHg)		SBP error (mmHg)		DBP error (mmHg)	
		Signals	# Subjects	N	SBP, Mean±SD	DBP, Mean±SD	SDE	MAE	SDE	MAE
(8)	BP-Net	ECG, PPG, ABP	293	–${}^{a}$	–	–	3.58	2.59	1.97	1.33
(13)	MIMIC-II	PPG, ABP	92	∼18,000${}^{b}$	–	–	2.99	2.29	2.32	1.93
(14)	UCI	ECG, PPG, ABP	942	200,159	132.61±21.70	63.71±9.98	0.94	2.33	2.88	0.71
(15)	UCI	PPG, ABP	–	57,757	118.22±18.01	64.34±9.66	8.46	6.17	5.36	3.66
(5)	MIMIC-III matched subset	PPG, ABP	100	∼360,000${}^{b}$	–	–	4.56	3.52	2.82	2.20
(6)	MIMIC	ECG, PPG, ABP	48	∼1,152${}^{b}$	–	–	1.60	1.20	1.30	1.00
(16)	MIMIC + MIMIC-III	PPG, ABP	100	∼340,000${}^{b}$	–	–	4.42	3.68	2.92	1.97
(9)	MIMIC-III matched subset	PPG, ABP	1,131	6,478	130.84±20.27	64.48±9.51	15.67	12.08	7.32	5.56
(17)	VitalDB	PPG, ABP	1,567	126,327	115.40±13.70	61.40±7.40	11.56	–	6.52	–
(18)	MIMIC-II	PPG, ABP	500	9,000	–	–	4.74	3.23	1.96	1.59
(11)	MIMIC-II	PPG, ABP	304	106,074	–	–	6.69	5.95	3.97	3.41
(7)	UCI	PPG, ABP	1,557	574,900	–	–	5.41	2.30	5.65	3.97
(19)	MIMIC-III	ECG, ABP	1,711	∼299,000${}^{b}$	135.69±20.57	71.72±8.84	9.99	7.10	6.29	4.61
(10)	MIMIC-II	ECG, PPG, ABP	604	120,684	125.30±15.70	72.30±10.80	2.76	3.09	2.00	2.11
(20)	UCI	ECG, PPG, ABP	942	–	–	–	5.54	5.32	3.82	3.38
(12)	MIMIC-III	PPG, ABP	510	∼504,000${}^{b}$	–	–	–	9.43	–	6.88
(21)	MIMIC	ECG, PPG, ABP	39	–	–	–	1.26	0.93	0.73	0.52

ECG, electrocardiogram; PPG, photoplethysmogram; ABP, arterial blood pressure; N, number of BP estimations to be made from the dataset; SD, standard deviation; SDE, standard deviation of error; MAE, mean absolute error.

${}^{a}$Information is not available in the publications and cannot be approximated using other information.

${}^{b}$Information is not available in the publications, but is approximated using other information.

**Figure 1:**
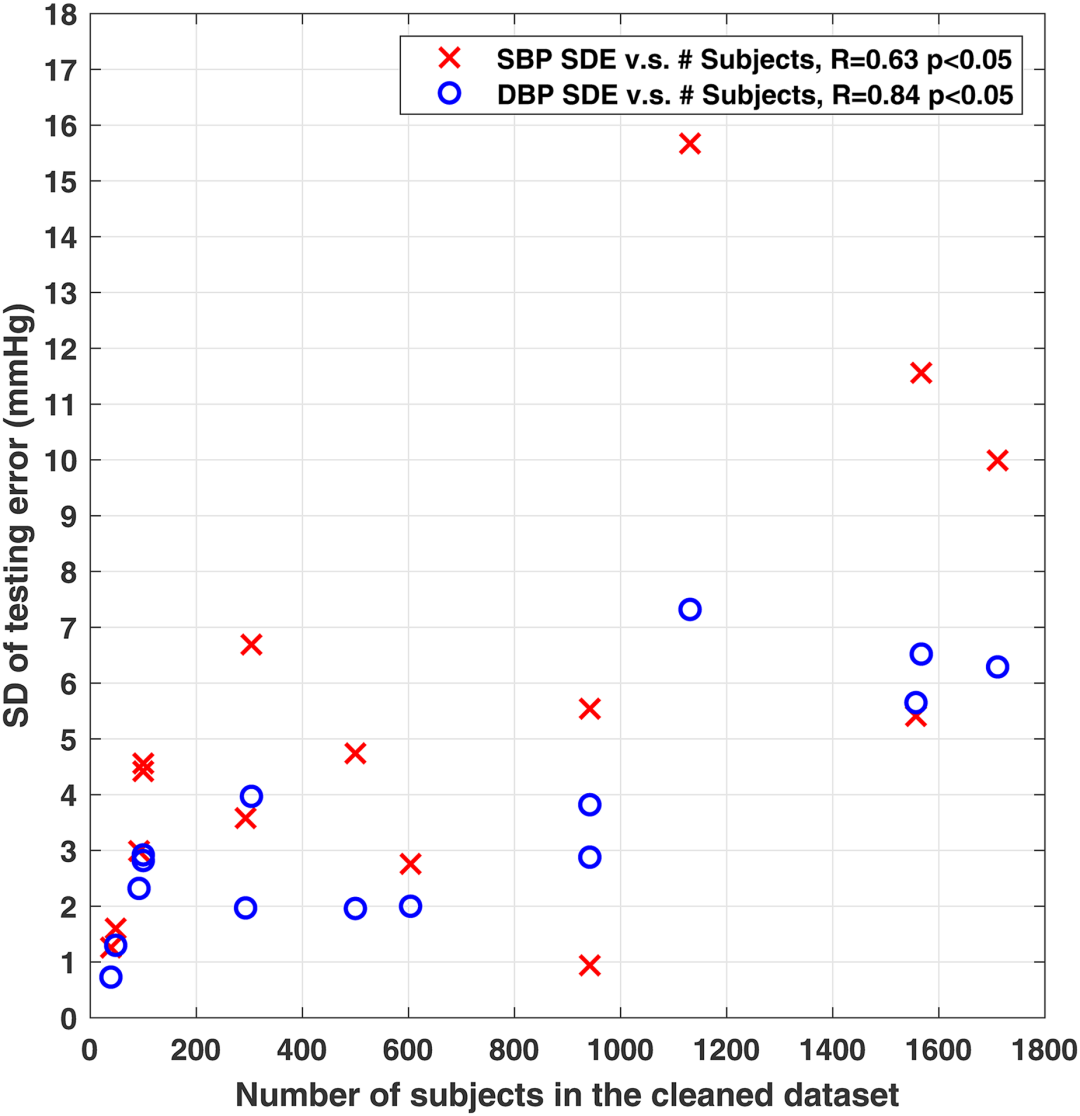
Scatter plot showing significant correlation between the reported standard deviation of error (SDE) of SBP and DBP, and the used number of subjects from studies summarized in Table 1. 15 of the 17 studies in Table 1 with reported number of subjects and SDE were included here.

An important point to observe from Table 1 is that while these studies have used publically-available databases (e.g., Multi-parameter Intelligent Monitoring for Intensive Care (MIMIC), MIMIC-II and MIMIC-III (22–24)), there exist significant differences in data selection criteria, pre-processing procedures, the number of included subjects, and the variations in reference systolic BP (SBP) and diastolic BP (DBP) in the datasets used for training and testing the models, across them. As the performance of deep-learning based BP estimation models is dependent on the characteristics of the dataset that is used for training and testing the model, such differences would make performance comparisons across models largely unfair. To further clarify this point, Figure 1 shows the correlation between the reported standard deviation of error (SDE) of estimated SBP and DBP, and the number of considered subjects, from the studies summarized in Table 1 that had reported both values. It can be seen that these two measures show correlation (moderate for SBP and high for DBP), suggesting that the BP accuracy metrics are impacted by the number of subjects used for training and testing the models. Therefore, having one unified benchmark dataset for computing performance metrics of BP estimation models is of need, in order to fairly compare the performances of the models and evaluate their generalizability.

To address the need of having a unified benchmark dataset, a few cleaned sources, which are subsets generated from publically-available databases, exist. These sources include the Cuff-Less Blood Pressure Estimation Data Set from the University of California Irvine (UCI) machine learning repository (25, 26) generated from the MIMIC-II database, and the BP-Net dataset (8, 27) generated from the MIMIC and MIMIC-II databases. However, they have limitations.

The UCI dataset lacks subject identification information, and consequently, it remains unclear how many subjects exist in the dataset, and which records belong to which subject. Indeed, there has been discrepancies in the reported number of subjects from this dataset across studies (e.g., 942 in (14), 4,254 in (25) and 12,000 in (7, 28, 29)), not leading to a verifiable conclusion. The lack of such information would make it difficult to use this dataset for evaluating the generalization capability of the models to new subjects (i.e., calibration-free testing). The BP-Net dataset contains data from 293 subjects, which can be too small for training deep learning models, considering that the Association for the Advancement of Medical Instrumentation (AAMI) standard requires more than 85 subjects to be included in the testing set (30, 31). If the BP-Net dataset is used for validating a model under the AAMI standard in calibration-free way, then there will be at most 208 subjects left in the training set, which could be insufficient for training deep learning models that require massive data to generalize. Moreover, both the UCI and the BP-Net datasets consist of long continuous signal records, which need to be further divided into subsets of short segments to match the input size of deep learning models.

Motivated by addressing these issues, here, we present PulseDB, the largest cleaned dataset to date, consisting of ECG, PPG and arterial BP (ABP) waveforms retrieved from the MIMIC-III waveform database matched subset (32) and the VitalDB waveform database (33). PulseDB offers the following features:

∙ With 14,570 h of ECG, PPG and ABP waveforms (stored in 10-s segments) from 5,361 subjects, PulseDB is the largest cleaned dataset to date. The large size is essential for training deep learning models that generalize well.
∙ Subject’s ID and demographic information are included for every segment in the PulseDB dataset, making it easy to group waveforms in the dataset with respect to subjects for fulfilling the requirements of the testing protocols, such as the AAMI. Moreover, BP estimation machine learning models can use the demographic information (e.g., age and gender) as additional features to further improve their estimation accuracy.
∙ The AAMI standard, which is widely accepted for validating cuff-less BP estimation models, requires more than 5% of DBP measurements to be lower than 60 mmHg (30, 31). In contrast to previous studies, which had set the threshold of the reference DBP in their dataset to be over 60 mmHg (2, 7, 19, 28) (and hence, may not fulfill the statistical requirements of the reference SBP and DBP in the AAMI testing protocol), here, we use a new, comprehensive data cleaning procedure that yields a cleaned dataset without directly thresholding the BP values, thereby, enabling us to generate a testing subset that fully meets the requirements of the AAMI testing protocol. As such, PulseDB enables standardized, reliable, and reproducible validation of cuff-less BP estimation models.
∙ In addition to the physiological waveforms and subject information, every segment in the PulseDB dataset also includes additional information such as the positions of beat-to-beat characteristic points, and the corresponding reference SBP and DBP values. Hence, PulseDB is directly usable for testing a wide variety of BP estimation models with different input and output requirements, e.g., models taking fixed-length windows (5, 14, 16) or beat-to-beat windows (6, 15, 21).

The full availability of subject information in the PulseDB dataset enables us to generate multi-functional training and testing subsets that support various model testing approaches. Furthermore, we use this dataset to investigate the reasons of performance gap in BP estimation models when data in the training and testing sets share subjects (referred to as calibration-based testing approach), and when the model is tested on data from subjects not used in the training process (referred to as calibration-free testing approach). Majority of the data-driven BP estimation studies to date, have reported their results using the calibration-based testing approach, thereby, the generalizability of the models to new subjects is not known. A few studies (9, 10, 12, 20) that have reported results from both testing approaches suggest significant performance difference. Using the training and testing sets created from PulseDB, we further investigate this issue.

The rest of this paper is organized as follows. Section 2 describes the procedures of generating the PulseDB dataset. Section 3 summarizes the information about the generated PulseDB dataset, along with an example of using this dataset. Finally, Section 4 discusses the advantages that PulseDB offers over existing datasets, and concludes the paper.

## 2. Methods

All 5,245,454 10-s signal segments in the PulseDB dataset are available for download from the GitHub repository at (34), in the form of MATLAB structure arrays stored in 5,361 MATLAB data files, each corresponding to one subject in the dataset. Here, we describe how the dataset is formed and structured.

### 2.1. Original source selection

We referred to the list of open cardiovascular waveform databases summarized in (35) to select the original sources of data to work with. Among the listed databases, the MIMIC-II and MIMIC-III waveform databases and their matched subsets, the VitalDB waveform database, and the Pulse Wave DataBase fulfill the requirements of simultaneously containing ECG, PPG and ABP recordings, as well as including a large number of subjects (>1000). The Pulse Wave DataBase is an in-vitro database generated from a simulated cardiovascular model (36), and was thereby excluded. In the remaining databases, only the MIMIC-II and MIMIC-III waveform databases matched subsets and the VitalDB database contain subject identification and demographic information. Since the MIMIC-III matched subset is a superset of the MIMIC-II matched subset (32), the MIMIC-III matched subset (32) and the VitalDB database (33, 37) were selected as the original sources.

### 2.2. Record selection

The MIMIC-III matched subset v1.0 (32) includes 22,317 records from 10,282 patients who stayed in the critical care unit (ICU) of the Beth Israel Deaconess Medical Center. The VitalDB database (33) contains 6,388 records from 6,090 ICU patients who underwent surgeries in the Seoul National University Hospital. Records in these two datasets were checked and retrieved using the WaveForm DataBase (WFDB) toolbox (38), and the VitalDB Python library, under the following protocols:

∙ *Presence of ECG, PPG and ABP signals:* A record must simultaneously contain PPG, ABP, and lead-II ECG signals to be included in our dataset. The lead-II ECG was selected as it was available in both original datasets. In both datasets, the PPG signals were measured from the fingertip (39, 40).
∙ *No invalid samples:* Signal records in both original datasets contain invalid placeholder values (NaN). Therefore, we defined a valid interval in each record as an interval in which all the ECG, PPG and ABP signals have valid numerical sample values. For each record, a valid interval with the longest duration was retrieved. The duration of the kept valid intervals ranged between 10 s and 10 h.

The above record selection procedure resulted in retrieving 4,941 records from the MIMIC-III matched subset, and 3,458 records from the VitalDB database. Signals acquired from the VitalDB database were downsampled from 500 Hz to 125 Hz to have a consistent sampling rate with the signals from the MIMIC-III matched subset.

### 2.3. Retrieving demographic information

In the MIMIC-III matched subset and the VitalDB database, the records are matched with the patients from whom the signals are retrieved. In the MIMIC-III matched subset, each record is named with a subject ID and the date of the ICU admission. In the VitalDB database, each record corresponds to a case ID that can be matched with a subject ID. For each record in PulseDB, the subject IDs provided in these two original datasets were matched back to their corresponding clinical databases to retrieve demographic information. For the MIMIC-III matched subset, subjects’ age and gender were fetched from the MIMIC-III clinical database (24). For the VitalDB database, subjects’ age, gender, weight, height and body mass index (BMI) were fetched from an affiliated web API (37).

### 2.4. Extracting the characteristic points

The following characteristic points were extracted from the ECG, PPG and ABP signals for each record in the PulseDB dataset.

#### 2.4.1. ECG R-peaks

The R-peaks of the ECG signal were detected for each record using the Pan-Tompkins QRS detection algorithm (41). This algorithm features a backtracking mechanism to robustly locate the R-peaks by estimating a running heart rate from 8 previously detected R-peaks. Therefore, it functions optimally when applied to the whole ECG record.

#### 2.4.2. PPG systolic peaks and turning points

The systolic peaks of the PPG signal were extracted using Elgendi’s algorithm (42), and the turning points were located as the minima between every two consecutive systolic peaks. This algorithm works by applying moving average filters to the PPG signal to estimate the baseline level of the signal, locating the systolic and diastolic phases of each PPG cycle as the major fluctuations of the signal with larger amplitudes than the baseline level. The PPG signal was filtered with a 4th order Chebyshev-II filter at [0.5,8] Hz before presenting to the Elgendi’s algorithm, as suggested in previous studies (12, 16, 43–45) for optimally suppressing noise and baseline wandering, and preserving PPG morphology features.

#### 2.4.3. ABP systolic peaks and turning points

Due to the presence of unspecified inter-signal delays in the MIMIC-III waveform database (39), beat-to-beat SBP and DBP values were extracted from cycles of the ABP signal. Considering the morphological similarity between the PPG and the ABP signals, the beat-to-beat systolic peaks and turning points of ABP were extracted using exactly the same method as for the PPG signal, and the amplitudes of the systolic peaks and the turning points were used as the SBP and DBP values of each beat.

### 2.5. Data segmentation and cleaning

After extracting the characteristic points from the records, we selected high quality segments from the records to form the cleaned PulseDB dataset. Data selection is conducted by dividing each record into 10-s non-overlapping segments, and determining whether to include or discard each segment. The reference SBP and DBP values of each segment are thus defined as the average beat-to-beat SBP and DBP values within each segment, respectively. As such, the dataset is ready to be used for supervised training of end-to-end deep learning models that take less than 10 s of ECG and/or PPG signals as input, for estimating the SBP and DBP values of each segment.

The low quality segments were rejected in the PulseDB dataset under the following procedure:

#### 2.5.1. Excluding saturated and flatline signals

Segments with ECG, PPG or ABP signals having more than 3 consecutive samples of the same value equaling to the minimum or maximum amplitude within the segment, or more than 1 s of the same amplitude, were removed from the dataset. As suggested in (12), this procedure removes segments containing saturated or non-recording signals, which are invalid for BP estimation.

#### 2.5.2. Validation of PPG and ABP signals

In order to have reliable reference BP values for model training and testing, segments with ABP signals with abnormal morphology (e.g., influenced by noise or interference) and/or with unrealistic sample values (e.g., negative amplitudes) were excluded. For this process, a widely-used exclusion method is to apply threshold to the SBP and DBP values extracted from the ABP signal, with the DBP level often set to be higher than 60 mmHg (2, 7, 19, 28). However, using such a range makes it difficult to generate testing subsets that fully comply with the AAMI testing protocol, which requires more than 5% of the reference DBP values to be lower than 60 mmHg (30, 31).

To address this issue, motivated by the fact that properly recorded PPG and ABP signals with normal morphology should be highly correlated (46), we propose a segment selection method based on validating the PPG and ABP signals mutually. That is, segments containing ABP signal with abnormal morphology can be excluded by checking the correlation between the PPG and the ABP signals. The following procedure was implemented.

∙ *Cardiac cycle detection:* A segment is included in the dataset only when at least 1 cardiac cycle of ECG, PPG and ABP signal in the segment can be located from their extracted characteristic points.
∙ *PPG signal quality evaluation:* 5-s sliding windows were applied to the PPG signal in each 10-s segment. The segment is excluded from the dataset if the skewness signal quality index (sSQI) of the PPG signal in any window is <0, as suggested in (47).
∙ *PPG-ABP correlation analysis:* The PPG and the ABP signals are first aligned using a lag that yields the highest cross correlation between the two signals to compensate the phase difference of the two signals and the inter-signal misalignment in the MIMIC-III database (16, 39). Next, the Pearson’s correlation coefficient between the aligned signals is calculated, and the segment is rejected if the correlation coefficient is <0.9, as suggested in (46).

### 2.6. Training and testing subsets for various testing protocols

The cleaned PulseDB dataset was further separated into 1 training and 3 testing subsets ready to be used for reproducible evaluation of deep learning-based BP estimation models. Each of these 3 testing subsets corresponds to a specific testing protocol, which are named here as the “AAMI” testing set, the “calibration-based” testing set, and the “calibration-free” testing set. Additionally, an “AAMI calibration” set is generated for calibration-based testing under the AAMI protocol.

The AAMI testing set was generated from the PulseDB dataset, such that it fulfills the requirements of the AAMI standard including having more than 85 subjects, having at least 3 estimations from each subject, and having more than 5% of very high and low SBP and DBP measurements within the testing set. Table 2 summarizes the requirements of the AAMI standard and their corresponding values for the testing set generated from the PulseDB dataset, indicating that all the requirements are met. Segments that were not included in the AAMI testing set, but were from subjects included in the AAMI testing set, were gathered to form the AAMI calibration set.

**Table 2:** AAMI standard requirements and their corresponding values for the AAMI testing set generated from the PulseDB dataset.

Checked items	AAMI requirements	AAMI testing set
Number of subjects	≥85	242
Number of measurements per subject	≥3	3–14
Number of total measurements	≥255	1,340
Proportion of reference SBP ≤100 mmHg	≥5%	14.63%
Proportion of reference SBP ≥160 mmHg	≥5%	15.52%
Proportion of reference SBP ≥140 mmHg	≥20%	38.81%
Proportion of reference DBP ≤60 mmHg	≥5%	25.75%
Proportion of reference DBP ≥100 mmHg	≥5%	9.40%
Proportion of reference DBP ≥85 mmHg	≥20%	27.24%

**Figure 2:**
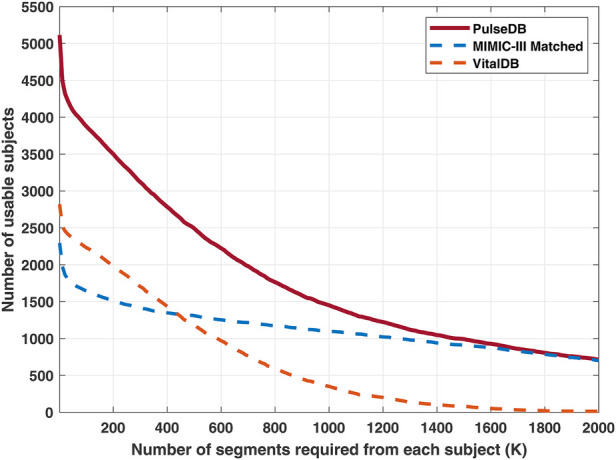
Red solid line: number of usable subjects in the PulseDB dataset when requiring each subject to have at least K segments. Blue and orange dashed lines: number of usable subjects from the MIMIC-III matched subset and the VitalDB database, for each selected K. The subjects used for generating the AAMI testing set were removed and not included here.

Next, all the segments from subjects who have been included in the AAMI testing and calibration sets were removed, and the remaining segments in the dataset were balanced with respect to each subject to ensure equal contribution from each subject. To achieve this, a desired number of segments (K) from each subject was selected. Figure 2 shows the trade-off between the number of segments required from each subject and the number of subjects having ≥K segments. Based on this, we selected K to be 400 to balance the number of subjects from the MIMIC-III matched subset and the VitalDB database. For subjects having more than K segments, K segments were randomly sampled from each subject, while other segments were discarded. From this subset, segments from 10% of randomly-selected subjects were drawn to form the calibration-free testing set. Segments from the remaining 90% of subjects were divided into the calibration-based testing set and the training set. For each of these subjects, 10% of segments were randomly sampled to be put into the calibration-based testing set, while the remaining segments form the training set. Thereby, a variety of testing sets (AAMI, calibration-free, and calibration-based) are readily accessible for evaluating the BP estimation models.

The GitHub repository (34) includes MATLAB scripts for reproducing the subject-balanced training set, calibration-based testing set and calibration-free testing set, as well as the AAMI testing set and the AAMI calibration set (see Table 4).

## 3. Results

### 3.1. Statistical information of the PulseDB dataset

**Figure 3:**
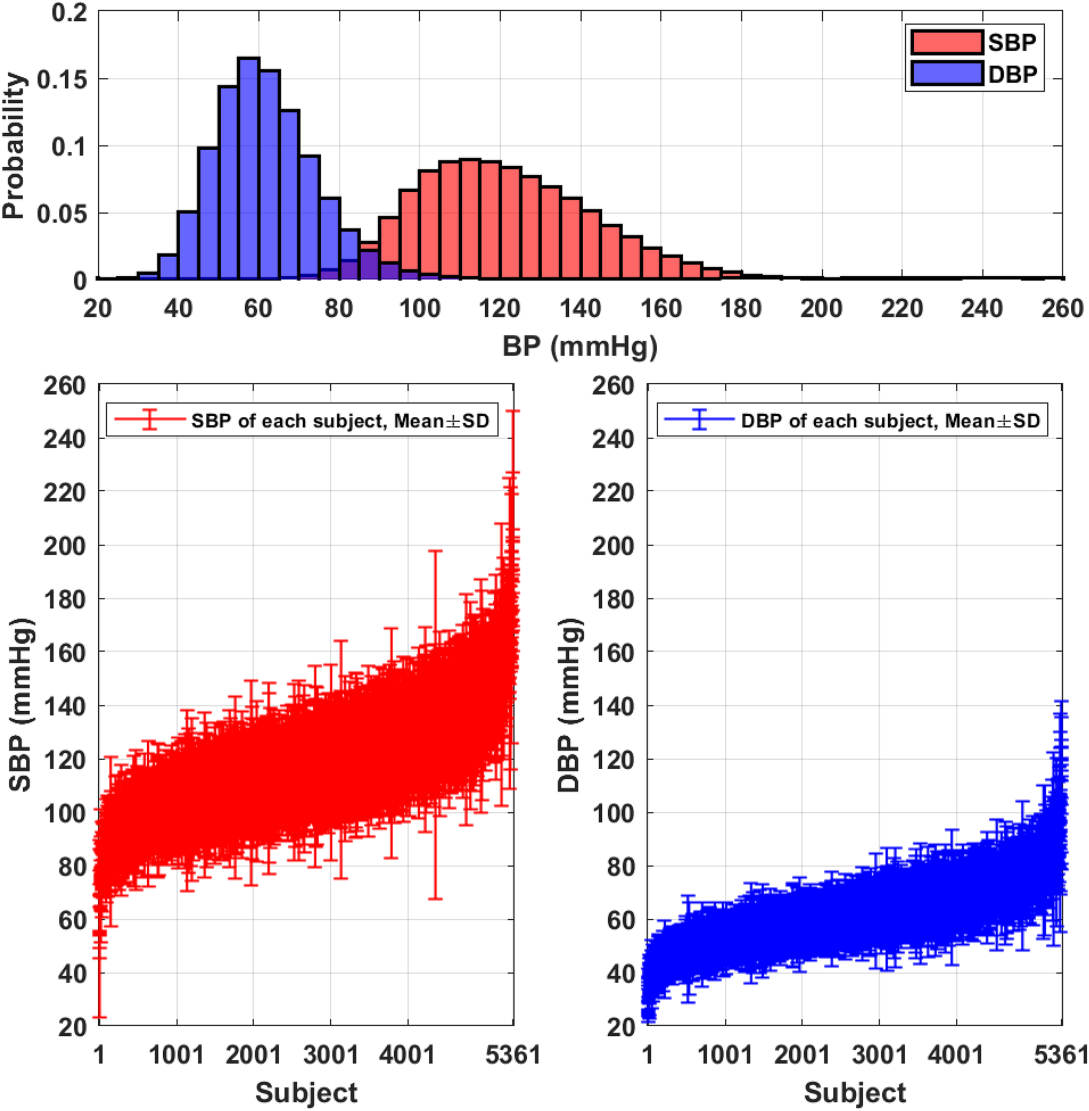
Distributions of the SBP and DBP in the PulseDB dataset. Top: histogram showing the distribution of reference SBP and DBP. Bottom: plot of subject-specific mean and standard deviation (SD) of SBP (left) and DBP (right).

Table 3 summarizes the demographic and BP distribution information of the proposed PulseDB dataset. The dataset includes 5,245,454 10-s segments from 5,361 subjects. The histogram of the SBP and DBP values and the mean and standard deviation (SD) of subject-specific SBP and DBP values in the PulseDB dataset are shown in Figure 3. As seen in Table 3 and Figure 3, the generated dataset is sufficiently large with respect to having both inter-subject and intra-subject variations in SBP and DBP, making it ideal for evaluating BP estimation model’s performance on tracking the BP changes of a subject, as well as estimating the baseline BP levels of various subjects.

**Table 3:** Summary of statistical information of the proposed PulseDB dataset.

Item	PulseDB
Number of subjects	5,361
Number of subjects from MIMIC/VitalDB	2,423/2,938
Number of segments	5,245,454
Age (mean ± SD)	60.87±15.58
Gender	3,013 male, 2,348 female
SBP (mmHg, mean ± SD)	121.42±22.10
DBP (mmHg, mean ± SD)	61.87±13.01
Average SBP per subject (mmHg, mean ± SD)	117.83±17.15
Average DBP per subject (mmHg, mean ± SD)	62.08±10.76
SD of SBP per subject (mmHg, mean ± SD)	11.87±6.26
SD of DBP per subject (mmHg, mean ± SD)	6.52±3.66

Table 4 summarizes the statistical information of the balanced training and 3 testing subsets, as well as the AAMI calibration set generated from PulseDB. As discussed, the training set and the calibration-based testing set contain segments from the same subjects in group A, and the AAMI testing set and the AAMI calibration set contain segments from the same subjects in group C (but with non-overlapping segments), while the training set, the calibration-free testing set and the AAMI testing set contain disjoint groups of subjects. As such, calibration-based or calibration-free testing can be achieved by choosing data from overlapped or disjoint groups of subjects for model training and testing (discussed further in Sections 3.3 and 4.2). Also, since the AAMI testing set requires specific ratio of very low and high SBP and DBP values, the SBP and DBP distributions in the manually-selected AAMI testing set are different compared to the other training and testing subsets that were generated from random sampling, with respect to having shifted mean values and larger SD.

**Table 4:** Summary of statistical information of the training, calibration, and testing sets generated from the PulseDB dataset.

Subset	Training	Calibration-based testing	Calibration-free testing	AAMI testing	AAMI calibration
Number of subjects	2,506	2,506	279	242	242
Subjects from MIMIC/VitalDB	1,213/1,293	1,213/1,293	135/144	126/116	126/116
Number of segments per subject	360	40	400	3–14	1–14,833
Subject group	A	A	B	C	C
SBP (mmHg, mean ± SD)	118.60±21.03	118.64±21.01	118.84±20.59	131.50±27.75	123.96±23.12
DBP (mmHg, mean ± SD)	61.86±12.65	61.88±12.64	62.00±12.27	73.61±18.22	63.18±13.63

### 3.2. Validation of signal quality of PPG and ECG segments

The effectiveness of the data cleaning procedure proposed in Section 2.5 was further validated by evaluating the quality of the ECG and PPG signals in PulseDB, using the method proposed in (48). This method judges the quality of the ECG and PPG signals using a combination of a set of feasibility rules and an adaptive template matching approach. The feasibility rules are defined as

1. The heart rate evaluated from the ECG R-peaks or the PPG systolic peaks in the 10-s segment should be between 40 and 180 beats per minute.
2. The maximum peak-to-peak interval of the ECG R-peaks or the PPG systolic peaks should be within 3 s.
3. The ratio of the maximum and minimum peak-to-peak intervals of the ECG R-peaks or the PPG systolic peaks should be less than 2.2.

If the feasibility rules are satisfied, then a signal quality index (SQI) based on adaptive template matching is calculated for each 10-s ECG or PPG segment as follow.

1. The median of beat-to-beat interval of the ECG R-peaks or the PPG systolic peaks is calculated as W.
2. Centering at each of the ECG R-peaks or the PPG systolic peaks, the ECG or the PPG windows with duration W are extracted. An adaptive template is calculated by averaging all windows taken from the segment.
3. The SQI of each 10-s segment is calculated as the average Pearson’s correlation coefficient between the adaptive template and each of the beat-to-beat windows within the segment.

It is suggested in (48) that ECG and PPG segments of good quality should have the SQI value exceeding 0.66 and 0.86, respectively.

The above described method was applied to all 5,245,454 segments in PulseDB, using the R-peak and systolic peak annotations extracted and included in PulseDB as described in Section 2.4. Results show that 94.3% of the segments have ECG signal satisfying the feasibility rules, among which 99.8% of the segments also satisfy the SQI threshold rule. Meanwhile, 95.9% of the segments have PPG signal satisfying the feasibility rules, with 99.7% of these segments also satisfying the SQI threshold rule. Considering the complicated physiological status of ICU patients involved in the PulseDB dataset, whose heart rate may fall out of the boundary defined by the feasibility rules (48), we can conclude that the ECG and PPG signals in PulseDB are of high quality.

### 3.3. Example of analysis with PulseDB: the performance gap between calibration-based and calibration-free model testing

BP estimation model studies have been typically using two approaches to evaluate the accuracy of their BP estimation models: “calibration-free” and “calibration-based.” Calibration-free approaches test the model with the data from subjects that are disjoint from the subjects used for training the model, while for the calibration-based approaches, the data in the training and testing sets share subjects in various ways, such as

∙ the training set is generated through random sampling over all data from all subjects, and the remaning data is used for testing,
∙ a set portion of data from every subject is used for training, and the remaining is used for testing,
∙ the model is trained using calibration-free approach, but is fine-tuned again using the data from testing subjects.

Some studies have considered both testing approaches, reporting a significant performance gap between the outcomes of the two. Table 5 summarizes the recent BP estimation studies that have included both calibration-based and calibration-free testing results of their proposed models. As can be seen, in all cases, compared to calibration-free, the models show lower error in calibration-based situations. The Pearson’s correlation coefficients reported in (10) shows a drastically reduced capability of tracking BP variation under the calibration-free testing protocol. The results suggest that these models have poor generalization to data from unseen subjects.

**Table 5:** Summary of studies comparing results from calibration-free and calibration-based testing approaches.

Citation	Model	Input	Calibration-free testing results							Calibration-based testing results						
			Testing set	SBP			DBP			Testing set	SBP			DBP		
				R	SDE	MAE	R	SDE	MAE		R	SD	MAE	R	SD	MAE
(9)	RNN	PPG-only	20% of subjects	0.44${}^{a}$	17.87	14.39	0.44${}^{a}$	8.43	6.57	20% of the entire dataset	0.62${}^{a}$	15.67	12.08	0.64${}^{a}$	7.32	5.56
(10)	CNN	PPG+ECG	10-fold cross validation over subjects	0.22	9.43	12.49	0.31	6.40	8.03	10-fold cross validation over the entire dataset	0.97	2.76	3.09	0.96	2.00	2.11
(20)	CNN	PPG+ECG	20% of records	–${}^{b}$	8.85	9.30	–	5.52	5.12	50% of testing data to fine-tune the model	–	5.54	5.32	–	3.82	3.38
(12)	CNN+RNN	PPG-only	Leave-one-subject-out	–	–	15.41	–	–	12.38	20% data from each leave-out subject to fine-tune the model	–	–	9.43	–	–	6.88

R, Pearson’s correlation coefficient; SDE, standard deviation of error (in mmHg); MAE, mean absolute error (in mmHg).

${}^{a}$Information is not available in the publications, but is approximated using other information.

${}^{b}$Information is not available in the publications and cannot be approximated using other information.

**Figure 4:**
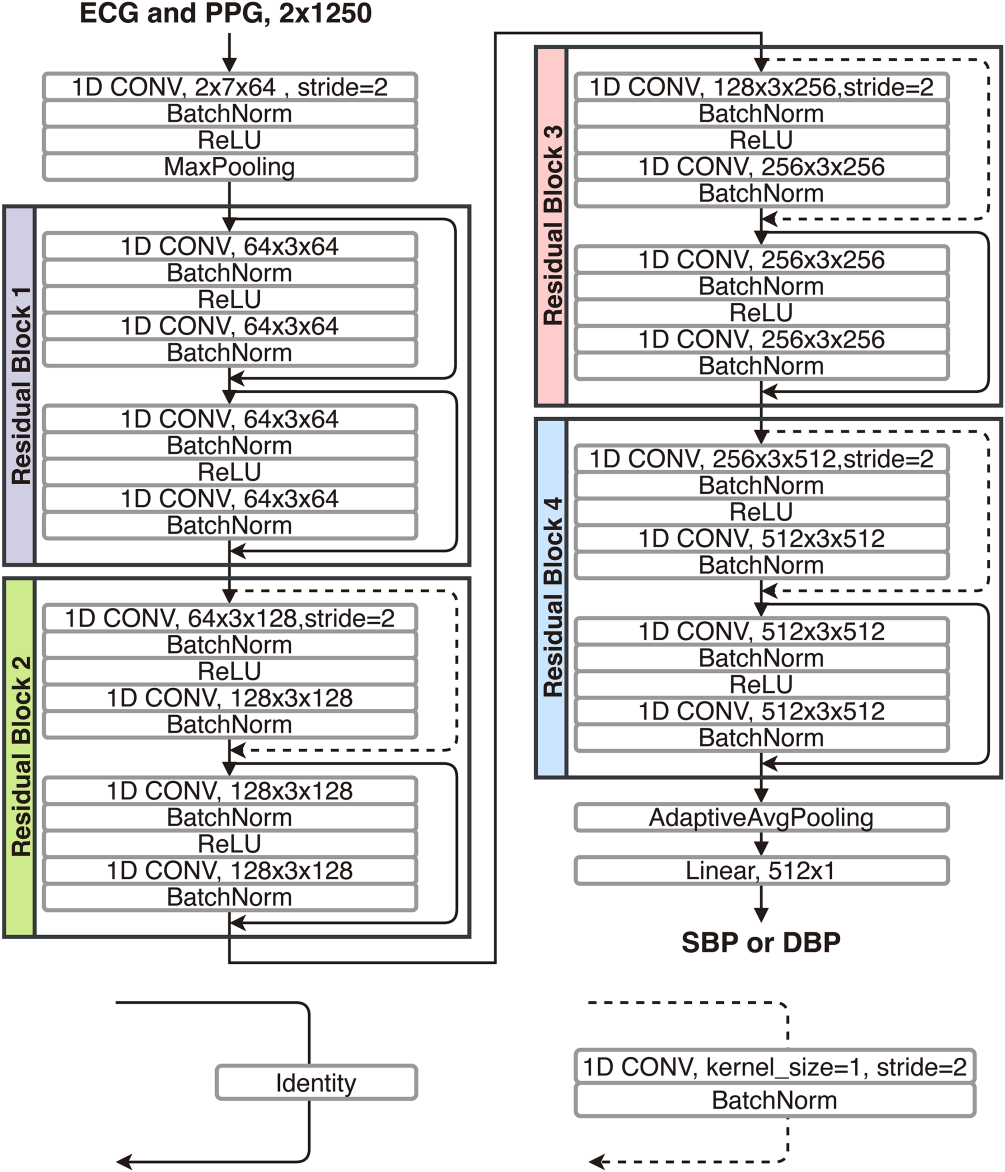
Structure of the 1D-modified ResNet-18 used for comparing calibration-based and calibration-free model testing approaches.

To further investigate the gap between the outcomes of the calibration-based and calibration-free testing approaches, here, using the training set and the two calibration-based and calibration-free testing sets from the PulseDB, we investigate the change of testing performances in the two testing approaches as the model gradually fits to the training set (49). We used a 1D-modified ResNet-18 (50) to estimate the SBP or DBP labels from the ECG and PPG signals in each segment. The architecture of the used 1D ResNet-18 is illustrated in Figure 4. The 1D ResNet-18 is constructed by replacing each of the 2D convolution, batch normalization and pooling layers in the original ResNet-18 design with their 1D substitutions, modifying the number of input channels to 2 for taking the ECG and PPG signals as inputs, and changing the final dense layer to have 1-dimensional regression output for BP estimation. Two models were trained, one for estimating SBP and one for estimating DBP, starting from the same parameter initialization with fixed random seed. Each model was trained for 100 epochs, using mean squared error (MSE) loss and Adam optimizer at $10^{-6}$ learning rate.

**Figure 5:**
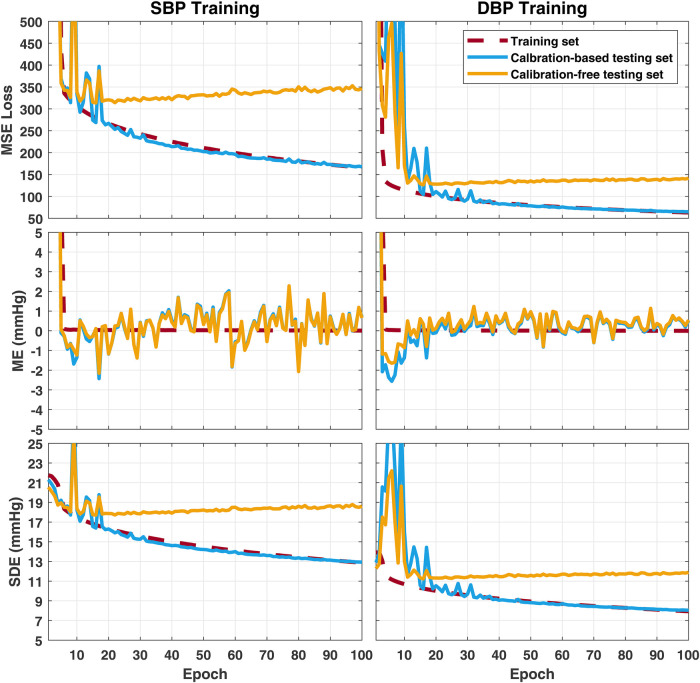
Change of error metrics on the training set, the calibration-based testing set, and the calibration-free testing set, when training the 1D ResNet-18 to estimate SBP (left) or DBP (right). MSE, mean squared error; ME, mean error; SDE, standard deviation of error.

Figure 5 summarizes changes of the MSE loss, the mean error (ME), and the SDE on the training set, the calibration-based testing set, and the calibration-free testing set, along the process of training the model epoch-by-epoch on the training set. Very similar trends were observed when the model is trained to estimate SBP or DBP. It can be observed that the ME on the training set is able to merge to 0 in the first few epochs, and remains at 0 for the rest of training epochs, while the SDE on the training set starts from being close to the SD of BP in the training set (as seen in Table 4), then, slowly decreases along the whole training process. This indicates that the model first estimates all output BP values with the mean BP value in the training set, then gradually learns to explain the BP variation in the training set using the information from the input. However, generalization is only observed on the calibration-based testing set, whose loss and SDE consistently decreases as the training goes on, while on the calibration-free testing set, a sign of over-fitting shows up early within the first 20 epochs, after which the loss and SDE start to increase instead of decrease. This implies that the additional information that the model learns after the first 20 epochs can improve the BP estimation accuracy on the group of subjects whose data has been seen by the model in the training set, but is not capable of explaining the BP variation on new, unseen subjects. Such incapability of generalization to data from unseen subjects leads to the model’s performance gap when validated using calibration-based and calibration-free testing, even when the two testing sets share similar BP distributions, as summarized in Table 4.

The lack of generalization on unseen subjects could be due to the heterogeneity of cardiovascular relationships between ECG, PPG and BP among people. Additional information such as subject’s demographics may be fused to the deep learning process to provide information that better explains the differences across subjects, which could have been hard to infer from the ECG and PPG signals alone. On the other hand, in the ECG and PPG signals, physiological information resides in the temporal scaling and shifting of recurring amplitude patterns caused by cardiac cycles. Therefore, the deep learning architectures for interpreting ECG and PPG signals may have to be optimized for learning from quasi-periodic signals, as well as for regression. Moreover, applying restrictions on the feature maps or embeddings extracted by the deep learning models, such as the usage of domain adversarial training (28) or generalizable independent latent excitation (51) can help enforcing subject non-specificity of the model. These methods may improve the cross-subject generalization capability of BP estimation models.

## 4. Discussion

### 4.1. Comparison with existing datasets

Table 6 compares the PulseDB dataset proposed here to other open datasets that have been used in cuff-less BP estimation studies. The PulseDB dataset offers the following advantages:

∙

*Inclusion of demographic information:* By choosing the MIMIC-III matched subset and the VitalDB database as the source datasets, the proposed PulseDB dataset includes demographic information, which would enable subsets generated from PulseDB to meet specific testing requirements, such as grouping subjects with respect to age or gender, or separating subjects that have been used for training from testing. Furthermore, the availability of age and demographic information in the dataset could be used as input information, in addition to the physiological signals, to improve the performance of the BP estimation models. On the other hand, in the MIMIC, MIMIC-II, MIMIC-III, and University of Queensland databases, physiological signals in the databases were archived as records, with each record corresponding to a consecutive running session of the bedside monitors in the operating room (39, 53, 54, 56). For the University of Queensland and the MIMIC databases, the demographic information was not recorded, while for the MIMIC-II and MIMIC-III databases, demographic information (age and gender) is available in stand-alone clinical databases. However, the ID of record in the waveform database and the ID of subjects in the clinical database were independent. As such, without additional unpublished information that have been used to create the MIMIC-II and MIMIC-III matched subsets, demographic information is not available for MIMIC, MIMIC-II, MIMIC-III and University of Queensland databases, as well as the UCI dataset and the BP-Net dataset that were derived from them.

∙

*Data cleaning:* As seen in Table 6, the PPG and ABP signals are not always available for all subjects in the MIMIC, MIMIC-II, MIMIC-III and VitalDB databases, as well as the MIMIC-II and MIMIC-III matched subsets. Moreover, records in these databases often contain segments with invalid signals, since sensors may be removed from the patients during the period in which the monitor was on. Consequently, these databases are not suitable for benchmarking BP estimation models before performing data cleaning procedures. This has led to the creation of cleaned databases such as the PulseDB, the UCI dataset, and the BP-Net dataset, among which PulseDB is the largest with respect to the total signal duration and the number of included subjects.

∙

*Training and testing separations:* PulseDB is the first open cuff-less BP estimation dataset that has been separated to subject-balanced training and testing sets, with each of the testing set corresponding to a commonly-used testing protocol. These pre-defined training and testing subsets make it easy to have comparable, reproducible and standardized evaluation of cuff-less BP estimation methods.

**Table 6:** Comparison between the proposed PulseDB dataset and other cardiovascular signal datasets that have been frequently used in cuff-less BP estimation studies.

Citation	Database	Source of data	# of subjects	Physiological signals	Demographic information
This work	PulseDB	MIMIC-III matched subset, VitalDB	5,361	ECG, PPG, ABP	Age, Gender, Height${}^{a}$, Weight${}^{a}$, BMI${}^{a}$
(25, 26)	Cuff-Less Blood Pressure Estimation Data Set (UCI)	MIMIC-II	–${}^{b}$	ECG, PPG, ABP	–
(8, 27)	BP-Net	MIMIC, MIMIC-II	293	ECG, PPG, SBP, DBP	–
(52)	MIMIC-II waveform database matched subset v3.1	MIMIC-II	2,809	ECG, PPG${}^{a}$, ABP${}^{a}$, RES${}^{a}$, Others${}^{a}$	Age, Gender
(32)	MIMIC-III waveform database matched subset v1.0	MIMIC-III	10,282	ECG, PPG${}^{a}$, ABP${}^{a}$, RES${}^{a}$, Others${}^{a}$	Age, Gender
(22, 52)	MIMIC database v1.0	ICU patients	∼90	ECG, PPG${}^{a}$, ABP${}^{a}$, RES${}^{a}$, Others${}^{a}$	–
(23, 54)	MIMIC-II waveform database v3.2	ICU patients	–	ECG, PPG${}^{a}$, ABP${}^{a}$, RES${}^{a}$, Others${}^{a}$	–
(24, 39)	MIMIC-III waveform database v1.0	ICU patients	∼30,000	ECG, PPG${}^{a}$, ABP${}^{a}$, RES${}^{a}$, Others${}^{a}$	–
(33, 37)	VitalDB	ICU patients	6,090	ECG, PPG${}^{a}$, ABP${}^{a}$, Others${}^{a}$	Age, Gender, Height, Weight, BMI
(55, 56)	University of Queensland Vital Signs Dataset	Anesthesia patients	32	ECG, PPG, ABP, CO2, EEG${}^{a}$, Others${}^{a}$	–

ECG, electrocardiogram; PPG, photoplethysmogram; ABP, arterial blood pressure; CO2, capnography; EEG, electroencephalogram; RES, respiratory.

${}^{a}$Data is not always available throughout the entire database.

${}^{b}$Information is not available in the database.

All 5,245,454 10-s signal segments in the PulseDB dataset are available for download from the GitHub repository at (34), in the form of MATLAB structure arrays stored in 5,361 MATLAB data files, each corresponding to one subject in the dataset. The GitHub repository also includes MATLAB scripts for reproducing the subject-balanced training set, calibration-based testing set, calibration-free testing set, and AAMI testing and calibration sets summarized in Table 4 from the segment files.

### 4.2. Protocols for benchmarking using PulseDB

PulseDB supports a number of protocols for benchmarking BP estimation models. Below, we discuss how different benchmarking protocols can be realized using the PulseDB subsets, and how BP estimation errors can be evaluated.

#### 4.2.1. General model training and testing

As seen in Table 4, the subject-balanced training, calibration-based testing and calibration-free testing sets of PulseDB share similar BP distribution, making them ideal for evaluating the performance of BP estimation models. Note that for methods summarized in Table 5, the calibration-based and calibration-free testing approaches require re-splitting the dataset into subsets, thereby, leading to different training data for each approach. However, in PulseDB, the subset generation methods (described in Section 2.6) enable the co-existence of calibration-based and calibration-free testing sets when the model is trained on single training set (as seen in Section 3.3). Using this feature of PulseDB makes it possible to record error metrics on both calibration-based and calibration-free testing sets simultaneously, for investigating the change in inter- and intra-subject generalization capability of the models upon training, parameter tuning, and structural adjustments.

#### 4.2.2. Training and testing under the AAMI protocol

By including the AAMI calibration set retrieved from the same group of subjects in the AAMI testing set, PulseDB supports model training and testing, subject to the AAMI protocol in both calibration-based and calibration-free manners. For a BP estimation model to demonstrate potential compliance with the AAMI standard, the trained model (either calibration-based or calibration-free) should be used to estimate one pair of SBP and DBP values for each segment in the AAMI testing set. For both SBP and DBP estimations, the ME and the SDE among all segments must be within ±5 and 8 mmHg (31).

#### 4.2.3. Error evaluation

A variety of error metrics can be evaluated for BP estimation, such as ME, SDE, the mean absolute error (MAE) (12, 14), the root mean squared error (RMSE) (8, 57), the unit-less coefficient of determination ($R^{2}$) (9, 58) and the Pearson’s correlation coefficient (R) (10, 16). Below, we discuss the significance of including these metrics for evaluating the performance of BP estimation models.

ME and SDE are calculated as

(1) $$\mathrm{ME}=\frac{1}{n}\times \sum_{i=1}^{n}({\hat{\mathrm{BP}}}_{i}-{\mathrm{BP}}_{i}),$$

(2) $$\mathrm{SDE}=\sqrt{\frac{1}{n-1}\times \sum_{i=1}^{n}[({\hat{\mathrm{BP}}}_{i}-{\mathrm{BP}}_{i})-\mathrm{ME}{]}^{2}},$$

where n is the number of total estimations, ${\mathrm{BP}}_{i}$ is the ith reference SBP or DBP value, and ${\hat{\mathrm{BP}}}_{i}$ is the ith SBP or DBP value estimated by the model. ME and SDE are estimators of the BP estimation bias, and the range of error in which the model’s error on the population resides, under the assumption of normally-distributed error (59, 60). However, limited data could impact the validity of these measures (31). For example, low ME and SDE values do not necessarily imply an accurate model when the SDE is close to the SD of reference BPs in the testing set. Therefore, it is important to interpret these metrics with information known about the dataset, such as the mean and SD of the reference BP values.

RMSE and MAE are commonly-used loss metrics, although many BP estimation models are trained with the MSE loss. The MAE is calculated as

(3) $$\mathrm{MAE}=\frac{1}{n}\times \sum_{i=1}^{n}|{\hat{\mathrm{BP}}}_{i}-{\mathrm{BP}}_{i}|.$$

Overall, we suggest reporting $R^{2}$ in addition to the above loss metrics to enable cross-dataset comparison of model’s performance, since it is an unit-less error metric normalized by the total sum of squares in the dataset, calculated as

(4) $$R^{2}=1-\frac{\sum_{i=1}^{n}({\mathrm{BP}}_{i}-{\hat{\mathrm{BP}}}_{i}{)}^{2}}{\sum_{i=1}^{n}{\left({\mathrm{BP}}_{i}-\frac{1}{n}\times \sum_{i=1}^{n}{\mathrm{BP}}_{i}\right)}^{2}}.$$

R, on the other hand, is not appropriate to be used as an error metric, since it depicts correlation instead of error, which can remain high even in the presence of large error (60).

### 4.3. Limitations

The ECG, PPG and ABP signals retrieved from the MIMIC-III database matched subset lack precise alignment, with an undetermined and inconsistent inter-signal misalignment for up to 500 ms (39). This limitation makes the analysis that rely on the synchronization of ECG, PPG or ABP waveforms (e.g., extraction of the pulse arrival time), unreliable. Unlike the MIMIC database, however, waveform synchronization is secured in the VitalDB dataset (33). Therefore, we suggest using segments from VitalDB for the analysis requiring precise inter-signal alignment among ECG, PPG or ABP.

To facilitate the usage of the PulseDB dataset for studies that rely on the alignment of ECG, PPG or ABP signals, a supplementary series of training, calibration-based testing, calibration-free testing, AAMI testing, and AAMI calibration subsets are generated, by choosing data only from the VitalDB subjects. Table 7 compares the statistical information of the supplementary AAMI testing set generated from 116 subjects of the VitalDB dataset with the AAMI standard requirements, confirming that all requirements are fulfilled. These subsets are available for download from Kaggle at (61), and can be reproduced from the script provided in the GitHub repository at (34).

**Table 7:** AAMI standard requirements and their corresponding values for the supplementary AAMI testing set generated from subjects retrieved from only VitalDB. This is a subset of the AAMI testing set summarized in Tables 2 and 4.

Checked items	AAMI requirements	Supplementary AAMI testing set
Number of subjects	≥85	116
Number of measurements per subject	≥3	3–10
Number of total measurements	≥255	666
Proportion of reference SBP ≤100 mmHg	≥5%	15.62%
Proportion of reference SBP ≥160 mmHg	≥5%	21.47%
Proportion of reference SBP ≥140 mmHg	≥20%	42.64%
Proportion of reference DBP ≤ 60 mmHg	≥5%	24.47%
Proportion of reference DBP ≥100 mmHg	≥5%	10.51%
Proportion of reference DBP ≥85 mmHg	≥20%	31.53%

### 4.4. Conclusions

In summary, we presented PulseDB, a new cleaned dataset for reliably validating deep learning-based cuff-less BP estimation methods. By combining signals from two publicly-available ICU databases, PulseDB is the largest cleaned dataset, to date, making it ideal for training and testing data-driven BP estimation models. The inclusion of subject’s demographic information and characteristic point positions in PulseDB extends its usage to various machine learning approaches, such as sequence-to-label estimation of SBP/DBP, sequence-to-sequence estimation of ABP, and beat-to-beat estimation of SBP/DBP, with no to minimal adjustment. The creation of the training set, the three testing sets (calibration-free, calibration-based and AAMI testing) enables reproducible and comparable accuracy evaluation across models, thereby, it can be used as a solid platform to fairly benchmark the generalization capability of BP estimation models and compare their estimation performance. An example study utilizing these subsets was conducted, which demonstrated that the performance gap between calibration-free and calibration-based model validation is due to the fact that information learned by the model, after the initial training epochs, generalizes to the group of subjects seen during training, but not to new, unseen subjects, probably due to the physiological differences across subjects. Overall, we expect the proposed PulseDB dataset to be a comprehensive, flexible and easy-to-use source of data for developing and evaluating cuff-less BP estimation methods.

### 4.5. Licenses

PulseDB is released with data derived from the MIMIC-III matched subset and the VitalDB dataset. Data derived from the MIMIC-III matched subset are released under the Open Database License (ODbL), while data derived from the VitalDB dataset are released under the Creative Commons Attribution-NonCommercial-ShareAlike 4.0 International (CC BY-NC-SA 4.0) license. Files and their corresponding licenses are specified in the GitHub repository of PulseDB at (34), as well as on Kaggle at (61).

## Data Availability

The original contributions presented in the study are included in the article/Supplementary Material, further inquiries can be directed to the corresponding author/s.
