# Supplementary material for: PulseDB: A large, cleaned dataset based on MIMIC-III and VitalDB for benchmarking cuff-less blood pressure estimation methods
Source: Front Digit Health. 2023 Feb 8;4:1090854. doi: 10.3389/fdgth.2022.1090854 (PMC9944565; doi:10.3389/fdgth.2022.1090854)
Supplement: Supplementary file 1 [file Datasheet1.pdf]

Supplementary Material

1 DATASET GUIDELINES

PulseDB is available for download from the GitHub repository at https://github.com/pulselabteam/PulseDB. The training, calibration, and testing subsets summarized in Table 4, as well as the supplementary training, calibration, and testing subsets described in Section 4.3 and Table 7, can be reproduced from the script provided in the GitHub repository. The supplementary subsets are also available from Kaggle at https://doi.org/10.34740/KAGGLE/DS/2447469. Here, we provide information about how to use this dataset.

1.1 File Structure

The PulseDB dataset is released as $5,361$ MATLAB® data files (MAT-File version 7.3), with each file corresponding to all segments belonging to one subject. The files were put into two folders, "**PulseDB_MIMIC**" and "**PulseDB_Vital**", separating subjects in the MIMIC-III matched subset from subjects in the VitalDB dataset.

Each of these files is an 1-D array of MATLAB structures. Each structure corresponds to a 10-s segment, which includes the following fields:

- **SubjectID**: It takes the format of "pXXXXXX", in which the 6 digits after "p" is the original subject ID used by the MIMIC-III matched subset and the VitalDB dataset. Segments in the same data file share the same **SubjectID**.
- **CaseID**: Identifier of record. Segments in the same data file with different **CaseID** come from different records belonging to the same subject.
- **SegmentID**: Sequence of segments. For segments with same **SubjectID** and **CaseID**, a segment with smaller **SegmentID** occurs prior to another segment with larger **SegmentID** temporally.
- **ECG_Raw**, **PPG_Raw**, **ABP_Raw**: Raw, unfiltered physiological signals directly cropped from records in the MIMIC-III matched subset and the VitalDB database. In each segment, the amplitude of **ECG_Raw** and **PPG_Raw** signals were linearly remapped between 0 and 1, while the amplitude of **ABP_Raw** signal was not modified, such that the absolute SBP and DBP values were preserved. These raw signals can be filtered with user-defined settings to be used as inputs or outputs that fit best to the desired BP estimation method.
- **ECG_F**, **PPG_F**, **ABP_F**: Filtered ECG, PPG and ABP signals. The ECG signal was band-pass filtered at $[0.5, 40]$ Hz with a Butterworth filter, while the PPG and ABP signals were band-pass filtered at $[0.5, 8]$ Hz using a Chebyshev-II filter, as described in Section 2.4. We suggest using the **ECG_F** and the **PPG_F** signals as inputs of BP estimation models, if there is no specific requirement for processing the input signals of the model.
- **ABP_Lag**, **PPG_ABP_Corr**: **ABP_Lag** is the lag that yields the highest cross correlation between **ABP_F** and **PPG_F**. The maximum possible lag is limited to $\pm$ 125 samples, or ± 1 s. The value of Pearson's correlation coefficient between **ABP_F** and **PPG_F** aligned using this lag is **PPG_ABP_Corr**.
- **ECG_RPeaks**, **PPG_SPeaks**, **PPG_Turns**, **ABP_SPeaks**, **ABP_Turns**: Positions of extracted characteristic points, specified as indices. For example, indexing **ECG_Raw** using **ECG_RPeaks** identify the amplitudes of all R-peaks of the ECG signal in this segment.

- **SegSBP**, **SegDBP**: the segment-averaged SBP and DBP values. We suggest using these values as reference SBP and DBP labels for training sequence-to-label BP estimation models (e.g. CNN), while using **ABP_Raw** for training sequence-to-sequence models (e.g. LSTM).
- **Age**, **Gender**, **Height**, **Weight**, **BMI**: Demographic information of the subject from which the segment was retrieved. The **Height**, **Weight** and **BMI** fields are only available for segments derived from the VitalDB database, since the MIMIC-III matched subset does not record these information.

1.2 Proxy Files for Statistical Analysis

To enhance data loading efficiency and to reduce memory cost for analyzing a large dataset, proxy files are generated for PulseDB to include only the demographic information and the reference SBP and DBP labels in each segment for basic statistical analysis, without requiring the effort of loading the waveform dataset.

Three types of proxy files are generated and included as part of the dataset. The first type, named as "**PulseDB_Info**", is a summary of demographic information and reference BP values for all subjects in the PulseDB dataset. The second type, named as "**Train_Info**", "**CalBased_Test_Info**", "**CalFree_Test_Info**","**AAMI_Test_Info**" and "**AAMI_Cal_Info**", corresponds to each of the training, calibration, and testing subsets of segments summarized in Table 4. Since these proxy files may record information of subjects from both MIMIC and VitalDB datasets, the field **Subj_Name** is used to identify different subjects, which consists of an additional digit of 0 or 1 after the **SubjectID** in the data files, to distinguish subjects in the MIMIC-III matched subset from subjects in the VitalDB dataset. Moreover, "**VitalDB_Train_Info**", "**VitalDB_CalBased_Test_Info**", "**VitalDB_CalFree_Test_Info**","**VitalDB_AAMI_Test_Info**" and "**VitalDB_AAMI_Cal_Info**" correspond to the supplementary subsets generated from only VitalDB subjects, described in Section 4.3 and Table 7.

1.3 Generation of Python-Friendly Training, Calibration and Testing Subsets

A MATLAB function is provided to generate the training, calibration, and testing subsets summarized in Tables 4, 7 and Section 4.3, using the subset separation settings defined in the proxy files. To maximize training and testing efficiency, the function fetches required data from the MATLAB data files that store the signal segments, and concatenates them into multi-dimensional arrays. These arrays can be effectively loaded into Python environments for training and evaluating deep learning models. The generated supplementary training, calibration, and testing subsets are available from Kaggle.
